# Transcervical fibroid ablation with the Sonata™ system for treatment of submucous and large uterine fibroids

**DOI:** 10.1002/ijgo.13638

**Published:** 2021-03-17

**Authors:** Gregory Shifrin, Matthias Engelhardt, Phyllis Gee, Gregor Pschadka

**Affiliations:** ^1^ Surgery of Tomorrow ASC Brooklyn NY USA; ^2^ Department of Gynecology Josephs‐Hospital Warendorf Warendorf Germany; ^3^ Willowbend Health and Wellness Frisco TX USA

**Keywords:** leiomyoma, radiofrequency ablation, SONATA, transcervical fibroid ablation, uterine fibroid

## Abstract

**Objective:**

To examine the role and benefits of transcervical fibroid ablation (TFA) in the treatment of submucous and large uterine fibroids.

**Methods:**

A subgroup of patients with submucous or large fibroids were analyzed from two prospective clinical trials (FAST‐EU and SONATA) of sonography‐guided TFA with the Sonata^®^ system. Key outcomes were changes in menstrual blood loss, symptom severity and health‐related quality of life on the Uterine Fibroid Symptom and Quality‐of‐Life Questionnaire, health‐related quality of life on the EQ‐5D questionnaire, and surgical reinterventions for heavy menstrual bleeding.

**Results:**

Among 197 women (534 treated fibroids), 86% of women with only submucous fibroids and 81% of women with large fibroids (>5 cm) experienced bleeding reduction within 3 months post‐ablation. Overall symptom severity and health‐related quality of life showed sustained, significant improvements over 12 months. Additional fibroid mapping of large fibroids with magnetic resonance imaging in the FAST‐EU trial showed an average volume reduction of 68%. Among women with only submucous fibroids, the rate of surgical reintervention through 1 year of follow up was 3.7% in FAST‐EU and 0.0% in SONATA.

**Conclusion:**

With the Sonata system, TFA is an effective single‐stage treatment option for non‐pedunculated submucous myomata, and larger or deeper uterine fibroids (including fibroid clusters) for which hysteroscopic treatment is not suitable.

**ClinicalTrials.gov:** FAST‐EU, NCT01226290; SONATA, NCT02228174.

## Synopsis

Transcervical fibroid ablation with the Sonata™ system is an effective single‐stage treatment option for non‐pedunculated submucous and large myomata.

## 1 INTRODUCTION

Uterine fibroids are highly prevalent, being present in up to 80% or more of women in the USA over their reproductive lives (depending on ethnicity).^1^ Several treatment options exist, but hysterectomy and myomectomy remain the most common interventions. Fibroid characteristics such as size, location, and number influence the choice and effectiveness of treatment. Using the FIGO (the International Federation of Gynecology & Obstetrics) classification, type 0–2 fibroids have submucous involvement, type 3 fibroids abut the endometrium but are completely intramural, type 4 fibroids are completely intramural, types 5 and 6 fibroids have serosal involvement, type 7 fibroids are pedunculated on the subserosal surface, and type 8 fibroids are identified in ectopic locations.^2^ With regard to submucous fibroids and large‐diameter fibroids, as there are several treatment options available, their optimal management may warrant further review.

Women with submucous fibroids (FIGO type 0, type 1, and type 2) are generally managed with operative hysteroscopy, including myomectomy and treatment with various morcellation devices. Although it permits transcervical management of such fibroids, this approach has limitations. Because visualization is via hysteroscopy, fibroids that are not visible from the endometrial cavity (e.g., intramural and subserous fibroids) are not typically amenable to hysteroscopic treatment. Larger and/or deeper type 2 submucous fibroids treated via hysteroscopic resection/morcellation often require a multistage approach because only a partial resection can be safely performed at the first operation. Subsequent interval extrusion of the remaining fibroid into the endometrial cavity permits additional resection later under hysteroscopic guidance.^3^, ^4^, ^5^ Emanuel et al.^6^ reported on their experience with hysteroscopic myomectomy in a cohort of 272 women where 17% of patients required multiple procedures to achieve complete myomectomy.

In a retrospective cohort study of 153 women undergoing hysteroscopic myomectomy, de novo intrauterine adhesiogenesis was detected in 1.5% of women with a solitary myoma, but 78% of women with at least two apposing myomata had intrauterine adhesions.^7^ Fluid overload, resulting in hyponatremia and the possibility of pulmonary and/or cerebral edema, is a potential complication for all operative hysteroscopy procedures, but is more common among patients undergoing operative hysteroscopy with monopolar electrosurgical systems.^8^

Transcervical fibroid ablation (TFA) with the Sonata^®^ system offers a less invasive, incisionless, uterus‐preserving, transcervical approach, with real‐time visualization using intrauterine ultrasound guidance. The system has been demonstrated as safe and effective for the treatment of symptomatic uterine fibroids.^9^, ^10^, ^11^, ^12^ It has received clearance in the USA by the US Food and Drug Administration as well as the Conformité Européenne Mark in the European Union. Because of its integrated high‐resolution intrauterine ultrasound probe, the Sonata system can visualize, target, and ablate all non‐pedunculated fibroid types. Sonata can treat a wide range of fibroid sizes and numbers with no specified limits. Multiple ablations can be used to successfully treat large fibroids if necessary. Specifically, the device can create scalable ablations ranging from 1 cm up to approximately 5 cm in maximum dimension (volume 42 cm^3^). This can be sufficient to treat a 5‐cm diameter myoma with a single ablation. Larger fibroids above 5 cm may require the application of multiple ablations. The Sonata system has been described in detail elsewhere.^12^, ^13^, ^14^

The prospective multicenter controlled FAST‐EU trial of TFA with the Sonata system required all patients to have at least one indenting fibroid (type 1, type 2 or type 2–5 [transmural] fibroids), and additional intramural fibroids were also treated if present.^9^ The trial followed patients through 12 months and reported significant improvements in menstrual bleeding symptoms and quality of life scores, low surgical reintervention rate, and no device‐related adverse events.

In the SONATA prospective multicenter controlled pivotal clinical trial of premenopausal women with symptomatic fibroids, women were required to have at least one indenting or abutting fibroid (type 1, type 2, type 2–5 [transmural], or type 3 [intramural] fibroids).^11^ The trial followed patients through 36 months and reported significant improvements in menstrual bleeding symptoms and quality of life scores, a low (5.5%) cumulative surgical reintervention rate, and no device‐related adverse events in the first 24 months.^10^, ^11^

In order to assess the clinical benefits of TFA when specifically used to treat patients with non‐pedunculated submucous (type 1 and type 2) uterine fibroids and contrast its role in the transcervical armamentarium, the clinical outcomes of prospective controlled trials involving patients with only submucous fibroids, as well as those with large fibroids over 5.0 cm in maximum diameter, were evaluated.

## 2 MATERIALS AND METHODS

Pooled individual participant data for this review were derived from two trials of TFA for symptomatic uterine fibroids, FAST‐EU and SONATA. The trial protocols are posted at ClinicalTrials.gov (FAST‐EU, NCT01226290; SONATA, NCT02228174). In each trial, patients provided written informed consent and ethics committees at each participating center provided study protocol approval. The FAST‐EU trial was a multicenter, prospective, controlled trial involving academic and community hospitals in the UK, the Netherlands, and Mexico.^9^ Fifty premenopausal women received TFA for heavy menstrual bleeding (HMB) as assessed by the Menstrual Pictogram with at least one indenting fibroid (type 1, type 2, or type 2–5 [transmural] fibroids), and additional fibroid types were also treated if present. The trial followed patients over 12 months, with specific imaging and other assessments performed at 3, 6, and 12 months. SONATA was a multicenter, prospective, controlled trial of sonography‐guided TFA with similar inclusion criteria and follow‐up assessments, with patient long‐term follow up for 3 years. Complete trial details have been previously reported for FAST‐EU^9^, ^13^ and SONATA.^10^, ^11^, ^15^ In this current subgroup analysis of individual participant data from both trials, we evaluated clinical outcomes in patients with submucous fibroids or large fibroids more than 5.0 cm in maximum diameter.

The sonography‐guided TFA procedure (Sonata system, Gynesonics, Inc., Redwood City, CA, USA) consists of an integrated intrauterine sonography probe and radiofrequency (RF) ablation handpiece that allows the gynecologist to identify, target, and ablate uterine fibroids. The integration of real‐time ultrasound imaging enables the physician to visualize, target, and ablate a greater range of fibroids than could be approached via operative hysteroscopy.^13^ A graphical interface is displayed on the live ultrasound image that identifies the target ablation area and the extent of thermal heating. The gynecologist uses this information to confirm that the ablation is within the fibroid while confining the thermal safety border to within the uterine serosa.

To ensure comparability of results between FAST‐EU and SONATA, data are reported through 1 year of follow up. Key outcomes included changes in menstrual blood loss, fibroid‐specific quality of life via the symptom severity score (SSS) and health‐related quality of life (HRQL) subscales of the Uterine Fibroid Symptom and Quality‐of‐Life (UFS‐QoL) Questionnaire,^16^ general health‐related quality of life via the EuroQol 5‐Dimension (EQ‐5D) questionnaire, and surgical reinterventions for HMB.

Data were reported using the mean and standard deviation for normally distributed continuous outcomes, median and interquartile range for non‐normally distributed continuous data, and count and frequency for categorical data. Reintervention due to HMB was analyzed using Kaplan‐Meier methods. Data were analyzed using SAS version 9.3 (SAS Institute, Cary, NC, USA).

## 3 RESULTS

In the FAST‐EU clinical trial, 50 women received TFA for the treatment of 92 fibroids. Of these 50 women, 42 had at least one submucous myoma and 10 women had at least one fibroid larger than 5 cm, with the largest measuring 6.9 cm in maximum diameter (Table 1). Additional fibroid mapping with magnetic resonance imaging in this trial showed an average volume reduction of 68.3% in fibroids larger than 5 cm. In the SONATA clinical trial, 147 women underwent a TFA procedure involving 442 fibroids; which 63 women had at least one submucous myoma and 9 women had at least one fibroid larger than 5 cm, with the largest measuring 6.5 cm in maximum diameter.

**TABLE 1:** Characteristics of treated fibroids in prospective trials of transcervical fibroid ablation^a^

Characteristic	FAST‐EU	SONATA
Number of patients	50	147
Number of treated fibroids	92	442
Treated fibroid type		
1	14 (15%)	15 (3%)
2	41(45%)	77 (17%)
2–5	8 (9%)	91 (21%)
3	4 (4%)	116 (26%)
4	25 (27%)	100 (23%)
5	0 (0%)	39 (9%)
6	0 (0%)	4 (1%)
Treated fibroid diameter		
Fibroids >5 cm	12 (13%)	11 (2.5%)
Patients with at least one fibroid >5 cm	10 (20%)	9 (6.1%)

^a^ Values are given as number (percentage).

**FIGURE 1:**
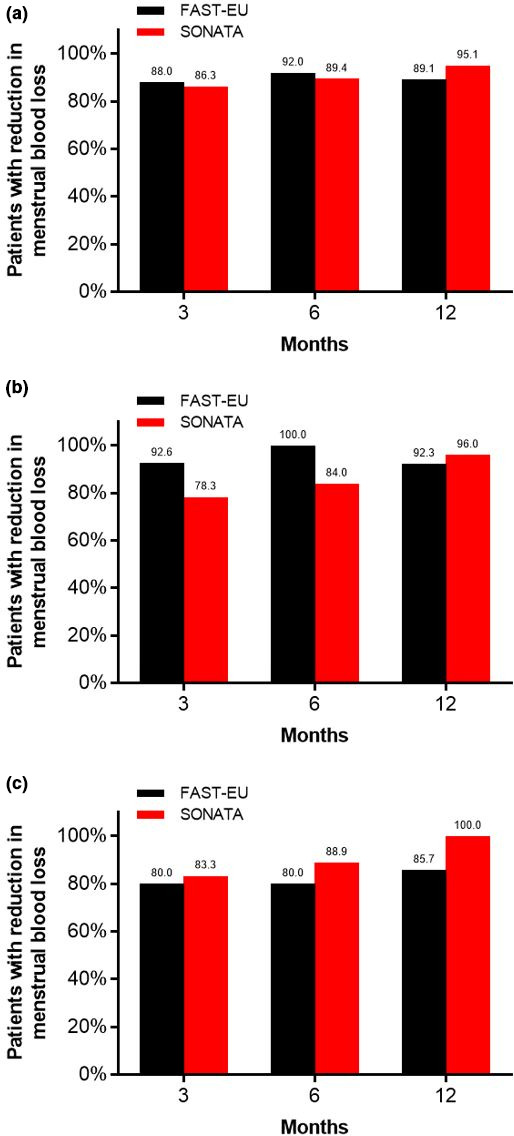
Percentage of patients with reductions in menstrual blood loss over 12 months after transcervical fibroid ablation in uterine fibroids among all patients (a), submucous fibroids (b), and large fibroids (c)

**FIGURE 2:**
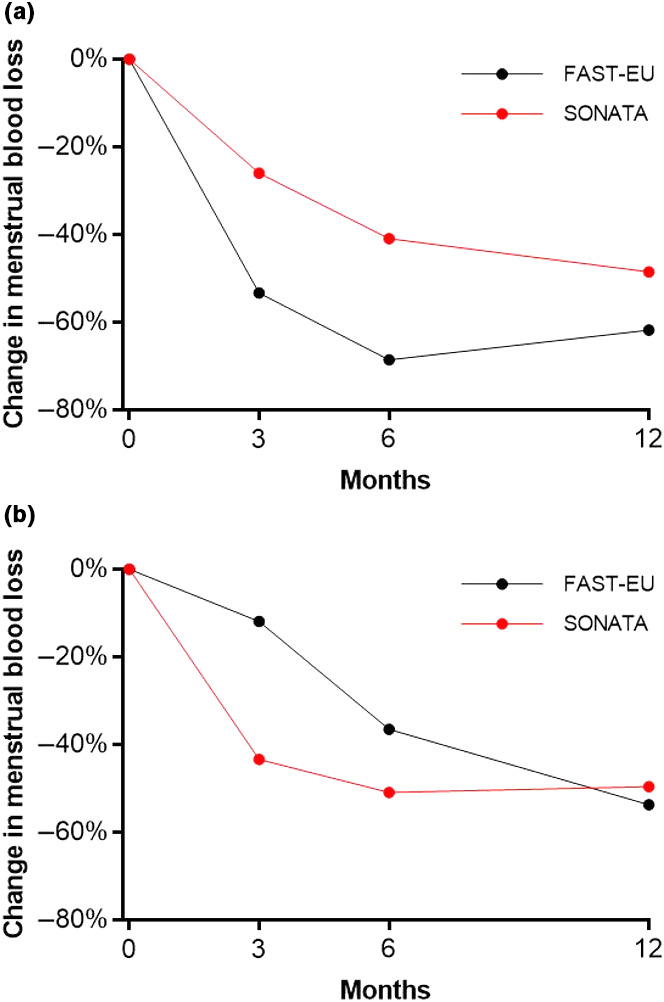
Change in menstrual blood loss over 12 months after transcervical fibroid ablation in submucous fibroids (a) and large fibroids (b)

**FIGURE 3:**
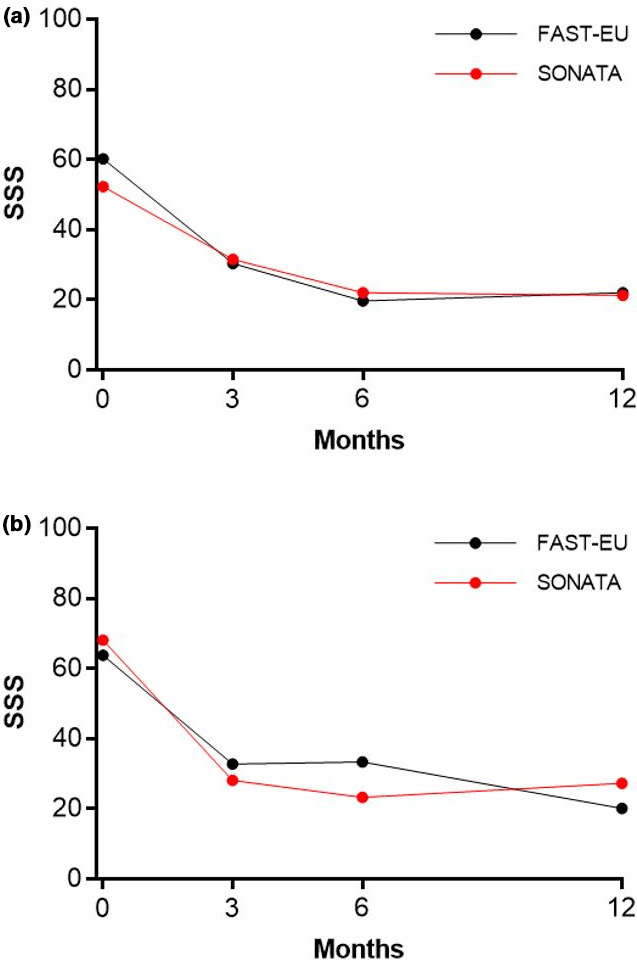
Change in Symptom Severity Score (SSS) over 12 months after transcervical fibroid ablation in submucous fibroids (a) and large fibroids (b)

**FIGURE 4:**
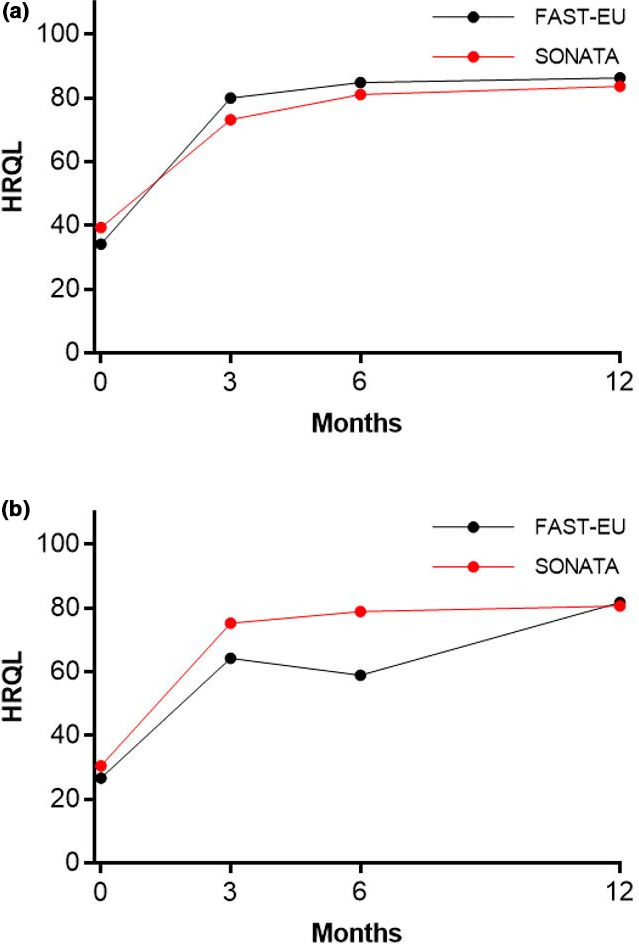
Change in Health‐related Quality of Life (HRQL) over 12 months after transcervical fibroid ablation in submucous fibroids (a) and large fibroids (b)

**FIGURE 5:**
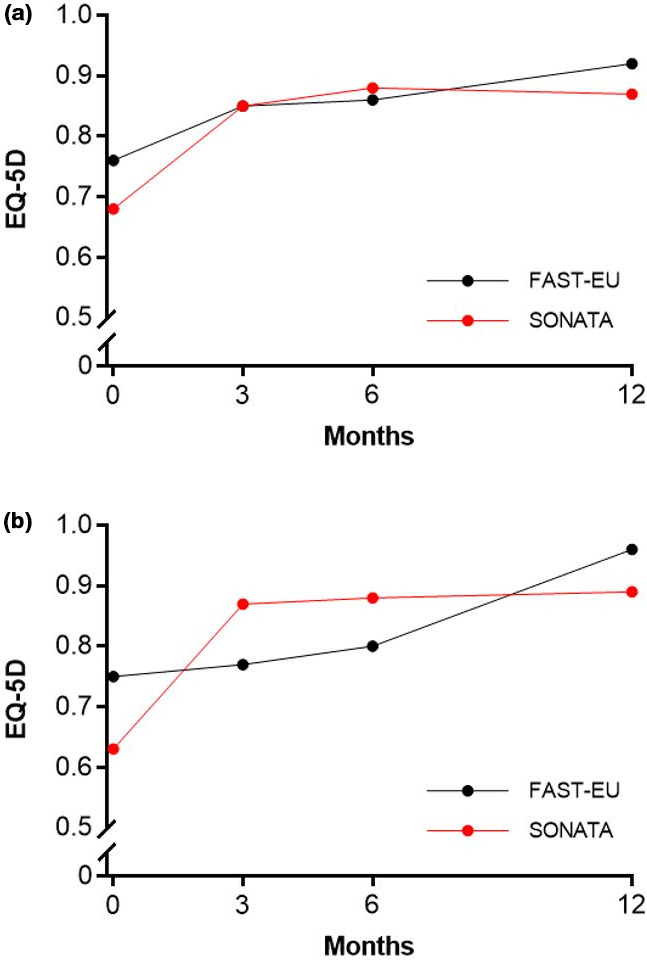
Change in general health‐related quality of life (measured as EuroQol 5‐Dimension [EQ‐5D]) over 12 months after transcervical fibroid ablation in submucous fibroids (a) and large fibroids (b)

In total, 72.5% of the 534 treated fibroids in both trials were not amenable to hysteroscopic resection because they were either intramural (45.9%), transmural (18.5%), or subserous (8.1%).^10^ Among all treated patients, TFA was highly effective in reducing menstrual bleeding. The percentages of patients with a reduction in menstrual bleeding at 3, 6, and 12 months were 88%, 92%, and 89%, respectively, in the FAST‐EU trial and 86%, 89%, and 95%, respectively, in the SONATA trial. The analysis in the report demonstrates similar trends in patients with only submucous fibroids and those with large fibroids (Figure 1). In these trials, improvements in menstrual blood loss were comparable in patients with only submucous fibroids and those with large‐diameter fibroids (Figure 2). The mean SSS decreased considerably within 3 months after TFA in each subgroup in these studies and was maintained through 1 year of follow up (Figure 3). Specifically, SSS improved by 39 points and 31 points at 12 months in patients with only submucous fibroids treated in the FAST‐EU and SONATA trials, respectively. Similarly, SSS improved by 35 points and 41 points at 12 months in patients with treated fibroids larger than 5 cm in the FAST‐EU and SONATA trials, respectively. Fibroid‐specific HRQL (Figure 4) and general health status on the EQ‐5D (Figure 5) rapidly improved in both subgroups at 3 months and was maintained over 1 year of follow up. Among women with submucous fibroids, the rates of surgical reintervention for HMB through 1 year was 3.7% in the FAST‐EU trial and 0.0% in the SONATA trial.

## 4 DISCUSSION

Transcervical fibroid ablation with the Sonata system offers a less invasive, incisionless fibroid treatment using intrauterine ultrasound guidance for visualization. It should not be thought of as the antiquated procedure known as “myolysis”. In myolysis, RF energy was used to create focal small ablations via multiple punctures through the uterine serosa without image guidance but with the potential for intra‐abdominal adhesiogenesis.^17^, ^18^ Myolysis was performed via either laparotomy or laparoscopy. In contrast, TFA enables an optimized volumetric ablation of a fibroid in which the ablated volume is tailored to that of the fibroid, thus minimizing the number of ablations. Additionally, TFA does not involve passage of electrodes through the serosa, is scalable, and is performed under real‐time integrated intrauterine sonography.

Clinical outcomes from both the FAST‐EU and SONATA clinical trials of TFA have demonstrated the safety and efficacy of the Sonata system for the treatment of symptomatic non‐pedunculated uterine fibroids, including intramural and subserous fibroids that are not amenable to operative hysteroscopy with either a resectoscope or transcervical morcellator.^9^, ^10^ In particular, this analysis demonstrates that TFA is effective in ameliorating symptoms and improving the quality of life in women with type 1 and type 2 fibroids as well as in women with large myomata.

In FAST‐EU, in which all patients had at least one indenting fibroid and 84% had at least one submucous fibroid, there were significant improvements in HMB as early as 3 months post‐TFA. Ninety‐three per‐cent of women with only submucous myomata and 80% of women with large myomata experienced a reduction in menstrual blood loss as early as 3 months post‐TFA. By 12 months, women in each subgroup experienced an average bleeding reduction of 62% and 54%, respectively. A 22% reduction in menstrual blood loss was shown by Lukes et al.^19^ to be the minimum reduction that is clinically meaningful to women, irrespective of baseline menstrual blood loss levels. Overall symptom severity and HRQL showed sustained significant improvements through 12 months post‐TFA in each subgroup.

The analysis of the SONATA clinical trial demonstrated similar efficacy outcomes of TFA for the treatment of submucous fibroids. In SONATA, which enrolled patients with at least one indenting (types 1, 2, 2–5) or abutting (type 3) fibroid, 43% of patients had at least one submucous fibroid. There were significant improvements in HMB as early as 3 months post‐TFA. In 78% of women with only submucous myomata and 83% of women with large myomata, a reduction in menstrual blood loss was experienced as early as 3 months post‐TFA. By 12 months post‐TFA, women in each subgroup experienced an average bleeding reduction of 48% and 50%, respectively. Similarly, overall symptom severity and HRQL showed sustained significant improvements through 12 months post‐TFA in each subgroup, with a low incidence of surgical reintervention during 12 months in both trials’ subgroups.

The ability to perform multiple ablations in a targeted fibroid enables the Sonata system to create a larger aggregated treatment volume. There are no specified limits to fibroid size or to the number of treatable fibroids with the Sonata system. In the SONATA clinical trial, as many as nine fibroids were ablated in a single patient; in some cases, a single ablation was used to treat one or more clusters of adjacent fibroids. Because of the orientation, proximity, and high resolution of the intrauterine ultrasound probe integrated within the treatment device, “single fibroids” visualized via transvaginal sonography may be seen to consist in actuality of clusters of two or more individual fibroids using intrauterine sonography. In such cases, it can be feasible to treat several clustered fibroids with a single ablation. Hence, the number and size of fibroids to be ablated is always up to a physician's clinical judgment.

Submucous fibroids are commonly managed with operative hysteroscopy, affording patients a transcervical option compared with open and laparoscopic myomectomy and hysterectomy. This technique is limited to the treatment of fibroids that are visible to the hysteroscopic surgeon, namely submucous myomata. In contrast, as demonstrated in both the FAST‐EU and SONATA trials, TFA can safely and effectively ablate non‐pedunculated submucous, intramural, transmural, and subserous fibroids. This allows physicians to treat the full range of non‐pedunculated fibroid types in the same session.^20^ Although hysteroscopic morcellators have been used to treat submucous fibroids, type 2 fibroids are a particular challenge for this approach, with a lower single‐stage success rate, a higher complication rate, and the recommendation that morcellators are not ideal for resection of type 2 fibroids.^21^ Because TFA does not involve significant uterine distension, fluid management systems are not necessary, nor is there a material risk of fluid overload, hyponatremia, and other attendant complications, unlike the situation with hysteroscopic resectoscopy and morcellation devices (e.g. TruClear, MyoSure, Symphion).^8^, ^12^, ^21^, ^22^ Patients typically do not need to return for additional treatment sessions after TFA, as all ablations are performed in the same treatment episode.

Kaplan‐Meier estimates of surgical reintervention rates after hysteroscopic management of uterine fibroids have been reported to be markedly greater than what is experienced after either abdominal or laparoscopic myomectomy. At 2 years, surgical reintervention was 18% after hysteroscopic myomectomy versus 8% for laparoscopic myomectomy and 6% for abdominal myomectomy; at 5 years, hysteroscopic myomectomy was associated with a 28% reintervention rate.^23^ The surgical reintervention rate for HMB at 2 years was 5.5% with TFA in the SONATA trial.^11^

Finally, TFA has not been associated with intrauterine adhesiogenesis. The OPEN clinical trial was a postmarket prospective, longitudinal, multicenter, interventional trial involving 37 women undergoing elective TFA with the Sonata system for symptomatic uterine fibroids.^20^ All women were required to have at least one indenting fibroid for inclusion in the trial, undergo baseline hysteroscopy with a second‐look hysteroscopy at 6 weeks post‐ablation to assess for the presence of de novo intrauterine adhesions, and have no evidence of intrauterine adhesions at baseline. Videos of all diagnostic hysteroscopies were assessed by independent reviewers and scored using the European Society of Hysteroscopy intrauterine adhesion classification system. Of 34 patients with evaluable baseline and second‐look hysteroscopies, none (0%) had any intrauterine adhesions at the 6‐week follow up according to the independent reviewers. Of note, six patients had apposing TFA‐treated fibroids without adhesiogenesis. This contrasts with a reported 78% risk of adhesions status after hysteroscopic myomectomy in apposing fibroids.^7^

This analysis is noteworthy for providing a broad collective analysis of women with fibroids who were treated in multiple countries by a variety of physicians. Despite the differences in patient and physician populations in both the FAST‐EU and SONATA clinical trials, the clinical utility of TFA with submucous and large fibroids was consistently demonstrated. The number of treated patients with large fibroids, however, is admittedly small. That reflects the reality that most fibroids in the population are no larger than 5 cm. Wegienka et al.^24^ evaluated 596 women with fibroids and found that 81.5% had fibroids of 5 cm or less in diameter; only 11.5% had large fibroids. Additional studies will likely reinforce the conclusions reached in this report.

In conclusion, data from prospective controlled clinical trials showed that TFA with the Sonata system is an effective single‐stage treatment option for non‐pedunculated submucous and large myomata. The number and size of fibroids to be treated are at the physician's discretion as there are no limits on the number or size of treatable fibroids with the Sonata system. Considering the disadvantages of hysteroscopic resection and morcellation of submucous fibroids, such as the need for staged procedures, a higher reintervention rate, and potential for intrauterine adhesiogenesis, TFA is a compelling option for the transcervical management of any non‐pedunculated, including submucous, myomata as well as larger, clustered, or deeper non‐pedunculated uterine fibroids for which hysteroscopic treatment is not suitable.

## CONFLICTS OF INTEREST

The authors have no conflicts of interest.

## AUTHOR CONTRIBUTIONS

GS, ME, PG, and GP contributed to the design, planning and conduct of the study and to writing the manuscript. Data analysis was performed by ABOND CRO, who was mentioned in the Acknowledgements section.
